# Hypnotism: Its History and Present Development

**Published:** 1890-02

**Authors:** 


**Hypnotism; Its History and Present Development.** By FREDRIK BJÖRNSTRÖM, M. D. Head Physician of the Stockholm Hospital; Professor of Psychiatry; late Royal Medical Swedish Councillor. Authorized translation from the second Swedish edition. By Baron Nils Posse, M. G. Director of the Boston School of Gymnastics. (Copyrighted by the Humboldt Publishing Co.) New York: 28 Lafayette place. Pamphlet, pp. 129.

This work of Björnström's is a careful *résumé* of the labor of the prominent hypnotisers, especially of the French schools. The subject is considered in its medical and legal bearings, and contains the report of many cases of cure—cases, we confess, that seem very startling, but which are vouched for by good observers. "They are all honorable men," but they do tell some mighty big stories. To the book is appended a bibliography of modern works. J. W. P.

